# Does Bleaching Affect the Microleakage of Class II Restoration with Bulk-Fill Composite?

**DOI:** 10.1155/2022/9924553

**Published:** 2022-01-05

**Authors:** Elham Zajkani, Mahdi Rahbar, Nima Motamed, Eissa Kordlou

**Affiliations:** ^1^Department of Restorative Dentistry, School of Dentistry, Zanjan University of Medical Sciences, Zanjan, Iran; ^2^Department of Restorative Dentistry, School of Dentistry, Ardabil University of Medical Sciences, Ardabil, Iran; ^3^Department of Community Medicine, School of Medicine, Zanjan University of Medical Sciences, Zanjan, Iran; ^4^Dentist, Zanjan, Iran

## Abstract

**Introduction:**

The use of technology in bulk-fill composites (BCs) has reduced the stresses caused by polymerization shrinkage, debonding, microleakage, or posttreatment sensitivity in them. This study was conducted to determine whether bleaching affects the microleakage of class II restoration with bulk-fill material.

**Materials and Methods:**

This laboratory study was performed on 40 normal human premolars in 4 groups (*n* = 20). Class II cavities were prepared in mesial and distal surfaces of the teeth with dimensions of 2 × 2 × 4 mm. Then, based on the bleaching process by 20% carbamide peroxide gel and using two types of composites, the restored cavities were randomly divided into 4 groups: (1) CC without bleaching (CC group), (2) BC without bleaching (BC group), (3) CC with bleaching (CCB group), and (4) BC with bleaching (BCB group). Then, the samples were thermocycled for 1000 cycles at a temperature range of 5–55°C, and they were immersed in 0.6% alkaline fuchsine in order to penetrate into the pigment for 24 h. After cutting, the samples were placed under a stereomicroscope (40%) to determine microleakage. The data were analyzed using one-way analysis of variance (ANOVA), and a *p* value <0.05 was considered as statistically significant.

**Results:**

Microleakage was determined in the CC group (0.97 ± 0.42), BC group (1.08 ± 0.54), CCB group (1.19 ± 0.37), and BCB group (0.30 ± 0.47). There were also no significant differences in the mean microleakage between the groups. No cases with zero microleakage (no microleakage) and grade 3 of microleakage (pigment penetration into the axial wall) were observed in the samples. Also, a two-by-two comparison of significant differences between CC and BC groups (*p*=0.89), CC and CCB groups (*p*=0.45), CC and BCB groups (*p*=0.11), BC and CCB groups (*p*=0.87), BC and BCB groups (*p*=0.41), and CCB and BCB groups (*p*=0.86) showed that the difference was not statistically significant.

**Conclusion:**

Results showed no difference between microleakage of BC and CC with and without bleaching, and bleaching had the same effect on microleakage of these two types of composites.

## 1. Introduction

Cosmetic dentistry is an important part of dental treatments, and the demand for it is increasing day by day [1]. Composite resins as the most common cosmetic restorative material used in the anterior part of the mouth best meet aesthetic and durability needs of restorations. Of course, composites have several undesirable properties that must be overcome to achieve long-term clinical success [2]. In composite restorations, microleakage of marginal area, failure of dental mass, reduction of light intensity entering material mass by increasing depth of composite material, and, consequently, insufficient cure depth are among the common problems [3]. Most problems of composite resin restorations can be directly or indirectly related to shrinkage during polymerization [4]. Volume shrinkage due to polymerization of the composites is about 2–6%, causing cracks in edges of the enamel and dentin, leading to the formation of a seam between the composite and tooth wall and weakening the connection. Formation of the seam causes microleakage, sensitivity, and recurrence of caries [2]. Microleakage around composite restorations means that microorganisms, saliva, molecules, and ions leak from the boundary between cavity walls and restorative material [4]. Microleakage occurs due to the following reasons: performing restoration process immediately after bleaching [5, 6], reaction between resin and peroxide remaining in the enamel [7], changes in the pH level [8], and application of the repeated pressures on restorative material [9].

One of the important requirements of cosmetic dentistry is the combination of bleaching treatments and the use of chemical agents to oxidize internal organic pigments by restorative treatments in order to achieve better color matching and beauty matching so that the effects of bleaching treatments have been considered before and after restorations due to clinical needs [9]. There are various reports on the effects of bleaching on composite resins [10]. Some reports have indicated a decrease in the surface hardness of the composite and formation of small cracks on it [11–13], and others have indicated its limited effects on the composites [1, 7, 14, 15]. Khoroshi et al. showed that performing light bleaching treatments immediately after restoration increased dentin microleakage of the restoration but did not influence microleakage of the same restorations at intervals of 1 week to 3 months [9]. Ellias and Sajjan demonstrated that the incidence of microleakage is increased by increasing bleaching time [16]. The evidence shows that the bleaching method is effective in reducing microleakage [7].

In the recent years, the use of bulk-fill composites in posterior teeth has increased due to new technology in their production [17]. These composites are able to restore cavities in thick layers with a thickness of 4–6 mm, and at the same time, they have the ability to maintain their mechanical properties in these thicknesses. In the new generation of bulk-fill composites, advanced monomer technology reduces shrinkage stress during polymerization and provides advantages, such as less cusp deflection in standard class II cavities and significant adaptation to cavities. It also prevents negative results of sensitivity after treatments, microleakage, and debonding. Besides, some studies have shown that the use of this composite has no effect on the occurrence of cervical microleakage [16, 18]. Given the existing inconsistencies in the previous findings and limited number of studies on microleakage of bulk-fill composites with and without bleaching, the present study was conducted to determine the amount of microleakage in bulk-fill composite restorations compared to the conventional composite in class II cavities with and without bleaching.

## 2. Materials and Methods

This experimental study was performed on 40 normal human premolars collected within 2 months and kept in normal saline solution. The teeth were all free of decay and cracks or other defects. After cleaning by using a scalpel and brush and applying pumice, the teeth were kept in Stimol 0.2 solution (Sigma-Aldrich Company, Tosol) for 24 h for disinfection, and then they were kept in distilled water for another 24 h.

The inclusion criterion was intactness of the premolars, and the teeth with caries, cracks, and other defects were excluded from the study. Also, the samples with fractures, pulpal exposure, and cracks during cutting were excluded from the study and were replaced with intact samples.

Class II standard cavities were drilled in mesial and distal surfaces of the teeth with 2 mm of buccolingual width, 4 mm of occlusogingival length, and 2 mm of mesiodistal width using a diamond cutter with 1 mm of diameter (Drendel + Zweiling, Germany) so that the gingival margin was 1 mm below the cementoenamel junction (CEJ). One type of cutter was used to cut all 4 holes.

**Figure 1:**
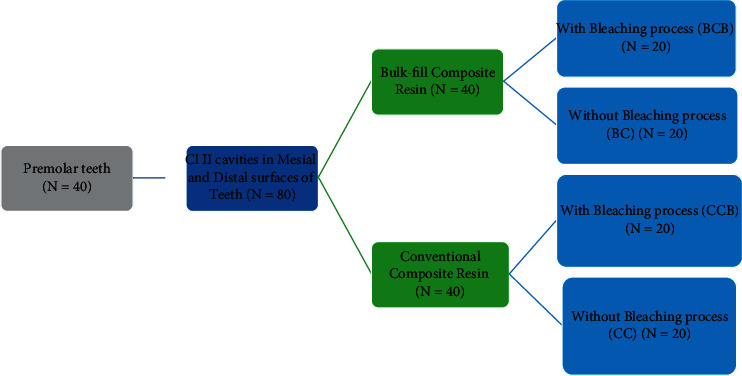
The study design (the materials and methods).

Groups were divided based on the bleaching process by 20% carbamide peroxide gel and composite type (*n* = 20) (Figure 1):

- CC group: conventional Z250 composite (3M, ESPE, USA) without bleaching
- BC group: Tetric EvoCeram (Ivoclar Vivadent Inc., Liechtenstein) bulk-fill composite without bleaching
- CCB group: conventional Z250 composite (3M, ESPE, USA) with bleaching
- BCB group: Tetric EvoCeram (Ivoclar Vivadent Inc., Liechtenstein) bulk-fill composite with bleaching

The samples in groups were restored by the conventional Z250 composite or Tetric EvoCeram bulk-fill composite according to the manufacturer's instructions. In CC and CCB groups, first, the cavities were etched using 37% phosphoric acid (3M, ESPE, USA) for 15 s and then were rinsed for 15 s, and they were dried by a gentle stream of air for 2 s (while the dentin was still moist). In the next step, Single Bond Adhesive (3M, ESPE, USA) was applied in two different layers in the samples, and they were exposed for 10 s. Mild air spray was used at the intervals between layers to evaporate the solvent. The Z250 composite was placed in the cavities of layers with 2 mm of thickness (layering technique) and was cured for 20 s (CC and CCB groups). The cavities of BC and BCB groups were prepared similar to CC and CCB groups, except that the Tetric EvoCeram bulk-fill composite was placed in the cavities of these groups according to the manufacturer's instructions with a thickness of 4 mm (bulky).

For exposing the restorations in all cavities, Bluephase C8 (Ivoclar Vivadent Inc., Liechtenstein) light-curing device with an intensity of 800 mW/cm^2^ was used for 15 s (according to the manufacturer's instructions), and after curing 5 samples, output intensity was examined by using a radiometer (Ivoclar Vivadent Inc., Liechtenstein). CCB and BCB groups were bleached in the study. In these groups, restorations were finished, and after performing a thermal cycle (at a temperature of 55℃ ± 5℃) for 30 s with 500 cycles and a rest time of 10 s using a thermocycling device (Vafaie, Iran), bleaching was done. In CC and BC groups, restorations were only finished, and thermal cycles were performed without bleaching.

For the bleaching process, 20% carbamide peroxide (Opalescence, Ultradent, USA, containing 20% carbamide peroxide gel and 0.11% potassium nitrate and fluoride ion) was used in equal amounts in each of the CCB and BCB groups for 14 days and 2 h a day daily (according to the manufacturer's instructions). For this purpose, the excess moisture of each sample was extracted using gas after extraction from artificial saliva and after each application of the whitening gel; the teeth were rinsed under running water for 2 min. At intervals between bleaching, the teeth were placed in an environment containing artificial saliva.

At the end of the bleaching period, the teeth were kept in water for 24 h, and then apex of all the samples was sealed using adhesive wax, and all tooth surfaces in all the samples except for 1 mm margin around the restorations were covered by 2 coating layers of nail polish in different colors. The samples were immersed in 0.6% alkaline fuchsine solution for 24 h, after which roots of the teeth were removed, and crowns of the teeth were cut in the mesiodistal direction and from the middle using the Mecatome machine (T201 A1, Presi, France).

In the next step, the degree of pigment penetration was evaluated using a stereomicroscope (Leica M, UK) with a magnification of 40x to determine the amount of penetration and compare the degree of microleakage, and then the results were recorded. Margin microleakage was determined using standard criteria:

- Grade 0: there is no microleakage
- Grade 1: pigment penetration about half the depth of the cavity
- Grade 2: pigment penetration more than half the depth of the cavity
- Grade 3: pigment penetration to the axial wall

According to the previous studies, the microleakage measurement method based on penetration of the pigment and observation under a stereomicroscope has both validity and reliability.

Data were entered into SPSS statistical software (version: 16) after assigning appropriate codes, and then they were analyzed. *p* value of 0.05 was considered as statistically significant for all tests.

## 3. Results

Initially, distribution of the data regarding microleakage in each of the 4 groups was checked using the Kolmogorov–Smirnov test, and the results showed that the data in all 4 groups follow a normal distribution. Because the significance level of this test was more than 0.05 in all groups, adherence to normal distribution in these groups was confirmed.

The mean ± standard deviation of microleakage in groups CC, BC, CCB, and BCB was obtained as 0.97 ± 0.42, 1.08 ± 0.54, 1.19 ± 0.37, and 1.30 ± 0.45, respectively. According to the results of one-way ANOVA, there was no significant difference in microleakage in the 4 groups including the groups that received bleaching, Z250 (conventional), and Tetric EvoCeram (bulk-fill) composites (*p*=0.138).

Also, a two-by-two comparison of significant differences between CC and BC groups (*p*=0.89), CC and CCB groups (*p*=0.45), CC and BCB groups (*p*=0.11), BC and CCB groups (*p*=0.87), BC and BCB groups (*p*=0.41), and CCB and BCB groups (*p*=0.86) showed that the difference was not statistically significant.

None of the studied groups presented grades 0 and 3 of microleakage. In other words, there was no case of microleakage or severe microleakage (pigment penetration to the axial wall) in any of the samples. However, frequency of microleakage grades of 1 and 2 in the CC group was equal to 9 (45%) and 11 (55%); in the BC group, it was equal to 11 (55%) and 9 (45%); in the CCB group, 8 (40%) and 12 cases (60%) were estimated, and in the BCB group, 6 (30%) and 14 cases (70%) were estimated. Results of the chi-square test showed no significant difference in the frequency of different degrees of microleakage in the studied groups (*p* < 0.05) (Table 1).

## 4. Discussion

Despite the increasing use of dental composites in modern dentistry, the use of these restorations is associated with problems, such as polymerization shrinkage and microleakage, which have reduced the success rate of these restorations. However, the type of composite used in terms of the amount of filler, the type of monomer, the degree of curing, and intensity of the optical hardener can all be effective in success of these restorations [18]. Along with cosmetic restorations, in the recent decades, bleaching (teeth whitening) treatment has received a great deal of attention, and this treatment can have different effects on the patients' restorations [19]. In this study, the degree of microleakage of class II bulk-fill composite resin restorations was investigated compared to conventional composites with and without bleaching conditions. According to the results of this study, microleakage was observed in all samples and groups, and no group was free of microleakage. On the contrary, no cases of severe microleakage were observed in any of the samples. In the present study, the highest mean microleakage levels were recorded in the groups that received the Tetric EvoCeram composite (with bleaching), Z250 composite (with bleaching), Tetric EvoCeram composite (without bleaching), and Z250 composite (without bleaching), respectively. Of course, mean microleakage was slightly different in different groups, but their differences were not statistically significant. Based on statistical and significance analyses of the results obtained from the study groups, bleaching factor and composite type had no effect on changes in microleakage in the samples.

Bleaching mechanism is based on the reaction of free radicals resulting from decomposition of hydrogen peroxide with pigmented carbon macromolecules [20]. Bleaching materials can pass through edges of the restorative material that is not well flooded or from porosities of the restorative material leading to microleakage [11]. Also, as bleaching time and concentration of the material are increased, surface porosity of the restoration is increased, and more surface sediments settle in it [21, 22]. Some studies have shown that the effects of teeth whitening on increasing the amount of fine leakage can be caused by peroxides deposited on the tooth surface and activated oxygen released by whitening agents preventing complete polymerization of the restorative material [23]. In addition, the increased microleakage following bleaching can occur due to hygroscopic increment after contact with the bleaching material and the increase in the toughness of the material, which influences bond strength, causing more microleakage in the treated samples [24, 25].

Herein, microleakage of class II restorations of the bulk-fill resin composite was investigated in comparison with the conventional composite with and without bleaching. According to results of this study, samples treated with bleaching showed more microleakage, but it was not statistically significant.

Consistent with the results of this study, Khoroushi and Fardashtaki investigated the effects of plasma arc bleaching on microleakage of class V restorations after restoration with the resin composite, compomer, and resin-reinforced glass ionomer and reported that bleaching with plasma arc has no effect on microleakage of tooth-colored restorations after restoration with Z100 resin composite, F2000 compomer, and Vitremer ionomer glass [26].

Klein et al. also investigated the effects of home and office bleaching agents on marginal microleakage rates of resin composite restorations and showed that the teeth that had not been treated with bleaching treatments had low marginal microleakage rates [27].

Contrary to our results, Ellias and Sajjan also experimentally investigated the degree of microleakage of resin composite in nonliving teeth and showed that bleaching had significant effects on seal quality in the area between the tooth and resin, and the degree of microleakage was also increased by increasing bleaching time [16].

Possible reasons for the difference between results of the previous studies and the present study could be the type of bleaching material, the type of composite, the type of the used adhesive, the type of tooth (human and bovine), the type of cavities (class II and class V), concentration of the material, and time of bleaching treatment.

Our results also showed no significant difference in the amount of microleakage between the conventional composite (Z250) used with the layering placement method (2 mm of thickness) and bulk-fill composite (Tetric EvoCeram) used with the mass placement method (4 mm of thickness).

Tetric EvoCeram is a bulk-fill composite containing a filler (78%) and a translucent matrix that helps light to pass through it. In this composite, a new optical initiator is used called as Ivocerin along with the conventional optical initiator, camphorquinone [28].

According to the manufacturer's instructions, this quick and complete curing starter is added to a depth of 4 mm. Ivocerin is a germanium-based primer that works as a supplement to camphorquinone. Optical absorption coefficient of Ivocerin is very high and can be effective in small volumetric amounts. Achieving low volumetric shrinkage stress is one of the primary and important goals in composite resins used as a mass in the cavity. In this regard, filler particles showing spring activity in modulating stress caused by polymerization have been added to this composite so that the stress caused by polymerization shrinkage of this composite in high volume and in a mass can be tolerated by the adhesive system [28].

In line with the results of our study, Sooraparaju et al. investigated microleakage of class V composite restorations including Tetric N-Ceram nanohybrid composite, Tetric N-Flow flowable composite, and G-aenial Universal Flo injectable composite and observed no significant difference between the groups in terms of microleakage in occlusal margins, but in the injectable composite group, they recorded less grade of microleakage in gingival margins, compared to other groups [29]. Also, no obvious differences were observed between the groups of conventional nanohybrid composite and flowable composite, so results of the two studies are comparable.

Ilie and Hickel and Campodonico et al. also showed that the amount of composite microleakage in the two methods of mass placement (bulk) and layering (layering) was not significantly different [18, 30].

Also, Mosharrafian et al. showed that marginal microleakage of the bulk-fill composite and conventional composite was not statistically significant [31].

Contrary to the results of this study, Alsagob et al. [4] showed that the amount of microleakage was higher in composite resin (flow) used with the mass placement method (4 mm) than the composite used with the layering placement method (2 mm of thickness). Among the possible reasons for this difference depending on the type of resin composite (flow) are the use of silver nitrate to investigate microleakage and differences in the type of bonding and the amount of thermal cycle (2000 cycles) [4].

Although laboratory studies are a rapid and relatively inexpensive method for obtaining information about the properties of restorative materials, such as microleakage and bond strength, however, the results of these studies cannot be generalized to clinical conditions in all aspects. There are many clinical variables, such as the extent of experience and skill of the practitioner in dealing with clinical conditions, procedure for the preparation of cavities, and hosting conditions in the patient's bed. Although laboratory studies have rarely shown a complete flood of restorative materials, most of them perform well in clinical settings. Some of the limitations of the study are difficulty in collecting intact samples, possibility of tooth damage during cavity preparation, lack of necessary equipment, and high costs of materials and equipment. While, in laboratory studies, efforts are made to standardize the conditions, however, it should be noted that the final evaluation of the performance of various restorative materials should be done through long-term and controlled clinical studies.

Future prospective is the use of various other resin composites to determine the degree of microleakage of restorations in comparison with different types of bulk-fill composite resins such as Tetric N-Ceram, determining the effects of bleaching on the bond strength of restorations, determining microleakage degrees of resin composite restorations by more accurate methods such as electron microscopy, determining the clinical and long-term results of using resin composite restorations of different types in preventing microleakage in restorations following bleaching treatments, and attempts to conduct research in clinical conditions.

## 5. Conclusion

The results of the present study showed that all the samples had some amounts of microleakage, and no statistically significant difference was observed in microleakage between bulk-fill and conventional composites with and without bleaching, and bleaching had the same effect on microleakage of these two composites.

## Figures and Tables

**Table 1:** Frequency and percentage of each degree of microleakage in the studied groups.

Groups	Degrees of microleakage	Frequency	The significance level
Group 1 (Z250 composite without bleaching)	0	0	0.476
	1	9 (45%)	
	2	11 (55%)	
	3	0	
Group 2 (Tetric EvoCeram composite without bleaching)	0	0	
	1	11 (55%)	
	2	9 (45%)	
	3	0	
Group 3 (Z250 composite with bleaching)	0	0	
	1	8 (40%)	
	2	12 (60%)	
	3	0	
Group 4 (Tetric EvoCeram composite with bleaching)	0	0	
	1	6 (30%)	
	2	14 (70%)	
	3	0	

## Data Availability

The data used to support the findings of this study are included within the article.
